# Modeling of the Long-Term Epidemic Dynamics of COVID-19 in the United States

**DOI:** 10.3390/ijerph18147594

**Published:** 2021-07-16

**Authors:** Derek Huang, Huanyu Tao, Qilong Wu, Sheng-You Huang, Yi Xiao

**Affiliations:** 1 Wuhan Britain-China School, No.10 Gutian Rd., Qiaokou District, Wuhan 430022, China; huangderek04@gmail.com; 2 Institute of Biophysics, School of Physics, Huazhong University of Science and Technology, Wuhan 430074, China; d201980099@hust.edu.cn (H.T.); m201970198@hust.edu.cn (Q.W.)

**Keywords:** COVID-19, epidemic model, transmission, epidemiology, vaccine prioritization

## Abstract

Coronavirus 2019 (COVID-19) is causing a severe pandemic that has resulted in millions of confirmed cases and deaths around the world. In the absence of effective drugs for treatment, non-pharmaceutical interventions are the most effective approaches to control the disease. Although some countries have the pandemic under control, all countries around the world, including the United States (US), are still in the process of controlling COVID-19, which calls for an effective epidemic model to describe the transmission dynamics of COVID-19. Meeting this need, we have extensively investigated the transmission dynamics of COVID-19 from 22 January 2020 to 14 February 2021 for the 50 states of the United States, which revealed the general principles underlying the spread of the virus in terms of intervention measures and demographic properties. We further proposed a time-dependent epidemic model, named T-SIR, to model the long-term transmission dynamics of COVID-19 in the US. It was shown in this paper that our T-SIR model could effectively model the epidemic dynamics of COVID-19 for all 50 states, which provided insights into the transmission dynamics of COVID-19 in the US. The present study will be valuable to help understand the epidemic dynamics of COVID-19 and thus help governments determine and implement effective intervention measures or vaccine prioritization to control the pandemic.

## 1. Introduction

Coronavirus disease 2019 (COVID-19) is a currently ongoing pandemic that has resulted in over 187 million confirmed cases and 4.04 million deaths globally [1]. Although several countries have the pandemic under relative control, all countries around the world, including the US, are still in the process of controlling the spread of COVID-19 [2]. In the US, a total of more than 27 million confirmed cases and over 480 thousand deaths have been reported as of 14 February 2021 [1]. Unfortunately, there are still no efficient antiviral drugs for the treatment of COVID-19. Vaccines and non-pharmaceutical interventions are therefore the only two available measures to control the transmission of the disease. As of 14 February 2021, more than 10 vaccines have been permitted by at least one national regulatory agency for distribution. However, current levels of vaccination are still insufficient to control the spread of the disease. In the US, over 97,000 new cases were reported on 14 February 2021 [1]. Therefore, government decisions on policies and interventions will be essential in controlling the spread of COVID-19 in the following months. Non-pharmaceutical interventional measures including social distancing, mandatory mask-wearing, isolation, and contact tracing are still crucial in controlling the spread of the disease [3,4,5,6,7,8,9,10,11].

Given the critical importance of non-pharmaceutical interventions in the control of COVID-19, modeling the past spread and predicting the future trends of the COVID-19 epidemic will play a vital role in decision-making by health officials regarding appropriate containment measures [12,13,14,15,16,17,18,19,20,21,22]. In the past year, many studies have been carried out to model or forecast the transmission dynamics of COVID-19 in various countries, regions, or cities around the world [23,24,25,26,27,28,29]. Among these, classical compartmental models have been widely used and obtained many successful predictions. Among different compartment models, the Susceptible-Infectious-Removed (SIR) model is the simplest model and provides the most primary transmission principles of all compartmental models [30]. Therefore, the SIR model and its extended versions have been widely used to describe the epidemic pattern of infectious diseases [31,32,33,34,35,36]. One basic parameter to describe the dynamics of an epidemic is the reproduction number, $R_{0}$, which represents the average number of secondary cases caused by one case in a large susceptible population. When $R_{0}>1$, an epidemic outbreak is expected to occur in a population, when $R_{0}<1$, the epidemic is under control, and when $R_{0}=1$, the epidemic is in relatively stable growth [31].

Traditionally, $R_{0}$ is assumed to be constant or arbitrarily change at specific times during the spread of a disease in compartmental models. This can be true for mildly infectious diseases like the flu [32], for which no preventive interventions are normally enforced by the government. However, this would not be the case for highly infectious diseases like COVID-19, for which no effective pharmaceutical remedies are available to control the disease. As mentioned before, many non-pharmaceutical interventions like mask-wearing, social distancing, prohibition of gathering, and school closure, have been enforced to control the spread of COVID-19. Therefore, with the development of the COVID-19 epidemic, the basic reproduction number $R_{0}$ is expected to gradually reduce due to the increasing number of preventive measures implemented over the course of the epidemic [37,38,39]. As such, the basic reproduction number $R_{0}$ will be time-dependent and should be represented by a dynamic value, $R_{t}$, which is defined as the average number of secondary infectious individuals generated by one infectious individual at time *t*. To incorporate such time-dependent dynamics, some studies have modified the traditional SIR compartmental model to track the early depleting transmission dynamics or forecast the relatively short-term trends of the COVID-19 epidemic [40,41]. Despite some successes using these models, they were only proposed for modeling the spread of COVID-19 in the early stages. These models would not be suitable for modeling the long-term transmission dynamics for an extended period of time. In addition, current models were only designed to predict the trends of epidemic situations with one outburst; however, situations with multiple outbursts cannot be predicted. Analysis of the data reported by different states in the US with high population densities shows a situation in which there is one outburst that is seemingly controlled, followed by a second outburst. The second outbursts occur at around the same time for each state. Therefore, previous models designed to predict only one outburst are unable to predict the epidemic trends in these areas. In addition, the epidemic dynamics of COVID-19 in the US are highly demography-dependent. Some states only show one apparent outbreak, while the others show two. Moreover, some outbreaks occurred in March–April 2020, but some occurred in June–August 2020, which would also be demographically dependent.

To address these issues, we propose a new time-dependent SIR model, named T-SIR, to model the transmission dynamics of COVID-19 from 22 January 2020 to 14 February 2021 for the 50 states in the US. Our T-SIR model provides a comprehensive picture of different scenarios of the long-term COVID-19 epidemic in the US. We analyzed the relationships between the epidemic parameters of our T-SIR model and the demographic properties of the states. The present study will be significant in predicting the future spread of COVID-19 at the state level. Utilizing this model, epidemic trends in certain areas can be used to help health officials make appropriate decisions on public health policies, including social distancing, wearing face masks, travel bans, lockdowns, and vaccine prioritization.

## 2. Methods

### 2.1. T-SIR Model

We have used the SIR compartmental model as the basis to build our time-dependent epidemic model for the spread of COVID-19. First, the SIR model is a classical compartmental model that was initially used to model the epidemic patterns of the flu. Despite its simplicity, the SIR model provides the most basic principles of transmission dynamics and has been widely used to model the spread of various infectious diseases [31]. Second, the SIR model is the simplest compartmental model and includes the fewest assumptions and a minimal number of parameters [40]. Therefore, the model would involve the least amount of overfitting problems possible and give a general description of the epidemic dynamics. Third, the SIR model has been used to model the transmission dynamics of COVID-19 in previous studies, which have shown the overall best fit for the early-time data of COVID-19 among different models including SIR, SEIR, and branching process models [25].

In the SIR model, individuals in a population are assigned to three compartments or categories: susceptible (S), infectious (I), and removed (R). Here, the S compartment stands for the susceptible individuals who are not immune to the virus and can become infected when they are exposed to the virus. Susceptible individuals cannot infect others because they have not carried the virus yet. The I compartment represents those individuals who are carrying the virus and infectious. The R compartment indicates those removed individuals who have either recovered or died from the disease and become immune to further infection. In this compartmental model, two important parameters determine the transmission dynamics of a disease. One is the transmission rate, β, which characterizes the probability of a susceptible individual becoming infected when the individual is exposed to an infectious individual during contact. The other is the removal rate, γ, which stands for the probability of an infectious individual becoming a removed individual during a specific time interval. For a population of N individuals, the SIR model can be described by the following set of differential equations [31]:

(1) $$\frac{dS}{dt}=-\beta \frac{IS}{N},\frac{dI}{dt}=\beta \frac{IS}{N}-\gamma I,\frac{dR}{dt}=\gamma I$$

where the total population, $N=S\left(t\right)+I\left(t\right)+R\left(t\right)$, remains a constant during the spread of the disease.

Since the time for an infectious individual to recover is relatively stable, the removal rate, γ, is often regarded as a constant. With these two parameters, β and γ, the basic reproduction number can be defined as $R_{0}=\beta /\gamma$. Due to the awareness-driven behavior and public health interventions in the spread of COVID-19, previous studies have shown that the transmission rate γ and corresponding reproduction number $R_{t}$ will gradually decrease over time. Therefore, considering the time-dependent trend in the transmission dynamics of COVID-19 and the multiple-outbreak feature of the COVID-19 epidemic in the US, we propose a dynamic epidemic model, named T-SIR, with a time-dependent transmission rate, $\beta \left(t\right)$, for modeling the spread of COVID-19 in the US as follows:

(2) $$\beta (t)=\left\{\begin{array}{c} \beta_{1}t^{-\sigma_{1}}\\ \gamma \\ \beta_{2}t^{-\sigma_{2}} \end{array}\right.\begin{array}{c} t_{0}<t\leq t_{1}\\ t_{1}<t\leq t_{2}\\ t>t_{2} \end{array}$$

where $\beta_{1}$ and $\beta_{2}$ stand for the initial transmission rate constants for the first and second outbreaks. The time t is in the unit of days and ranges in $\left[1,\infty \right]$. Between two outbreaks, there is a relatively stable stage with a reproduction number of $R_{t}\approx 1$, i.e., $\beta \left(t\right)\approx \gamma$. The parameter $\sigma_{1}$ and $\sigma_{2}$ are the corresponding exponents to describe the power decreasing feature of transmission rates and normally have a value between 0 and 1. Therefore, σ can be defined as the intervention parameter reflecting the force of intervention in the epidemic control of COVID-19. When σ=0, our time-dependent epidemic model T-SIR returns to the original SIR model. When σ is larger, the intervention force is stronger and the transmission rate $\beta \left(t\right)$ will reduce faster with time t. As the removal rate γ is assumed a constant in the model, the dynamic reproduction number $R_{t}=\beta \left(t\right)/\gamma$ will show a similar time-dependent relationship to the transmission rate $\beta \left(t\right)$. In this study, we will use our time-dependent SIR model, shown in Equations (1) and (2), to model the transmission dynamics of COVID-19 from 22 January 2020 to 14 February 2021 for the 50 states in the US.

### 2.2. Data Sources and Processing

#### 2.2.1. Epidemiological Data

Similar to the previous study [12], we obtained the COVID-19 epidemic data from the COVID-19 Data Repository managed by the Center for Systems Science and Engineering (CSSE) at Johns Hopkins University (JHU) [42]. First, the time series table file, “time_series_covid19_confirmed_US.csv”, was downloaded from the JHU CCSE COVID-19 Dataset website [42]. The time series data are updated once a day and includes the time series data of the cumulative daily confirmed cases from 22 January 2020 to 14 February 2021 in the 50 US states as of our download date. The original data, which is presented daily, reported the confirmed cases at the county level in each state. We then processed the data to obtain the total number of confirmed cases on different dates for each state by summing the corresponding data for all counties in the respective state. The processed data of confirmed cases can be freely accessed at https://github.com/HuangDerek/T-SIR (accessed on 3 July 2021).

#### 2.2.2. Geographical and Demographic Data

The most recent and accurate population data for each state were obtained from 1 July 2020 estimations from the US Census Bureau [43]. Total land area data were obtained from the US Census Bureau [44]. Population density data were obtained by calculations of the population divided by total land area for each state.

#### 2.2.3. Mobility Data

The mobility data table of the US states during the COVID-19 pandemic, named 2020_US_Region_Mobility_Report.csv, was first downloaded from the Google COVID-19 Community Mobility Reports website at https://www.google.com/covid19/mobility/ (accessed on 14 February 2021). The original region CSV table contains six types of mobility data including “retail_and_recreation”, “grocery_and_pharmacy”, “parks”, “transit_stations”, “workplaces”, and “residential” at the country, state, and county level, which are characterized by the “percent_change_from_baseline”. We then extracted the state-level mobility data from the table file because the present study focused on the epidemic dynamics of different states. Our examinations showed that the six types of mobility data are well correlated with each other. Therefore, for simplicity, we used the “retail_and_recreation” mobility data, which is also the most complete data in the table, to represent societal mobility during the COVID-19 pandemic. The processed mobility data for the different states can be freely accessed at https://github.com/HuangDerek/T-SIR (accessed on 3 July 2021).

### 2.3. Fitting

To accurately model the epidemic trends, the T-SIR model parameters, $\beta_{1}$, $\beta_{2}$, $\sigma_{1}$, $\sigma_{2}$_,_ and γ, are required to be determined by fitting the model with the cumulative data, where the corresponding dynamic reproduction numbers $R_{1}=\beta_{1}/\gamma$ and $R_{2}=\beta_{2}/\gamma$. We fitted our T-SIR model using the grid search of parameters $R_{1}$, $R_{2}$, $\sigma_{1}$, $\sigma_{2}$, and γ in reasonable ranges. The ranges for $R_{x}$, $\sigma_{x}$_,_ and γ were set to $\left[0.1,\;5\right]$, $\left[0,\;0.3\right]$, and $\left[0,\;0.3\right]$ with steps sized 0.1, 0.01, and 0.01, respectively [25]. The parameters of our T-SIR model, $R_{1}$, $R_{2}$, $\sigma_{1}$, $\sigma_{2}$, and γ, were then automatically determined through the best fit between the predicted data of T-SIR model and the reported COVID-19 data according to the mean squared error (MSE), where the three times, $t_{0}$, $t_{1}$, and $t_{2}$_,_ in Equation (2) were also automatically determined during the model fitting.

## 3. Results

### 3.1. COVID-19 Epidemics of the 50 States in the US

#### 3.1.1. Times of COVID-19 Outbreaks

**Figure 1:**
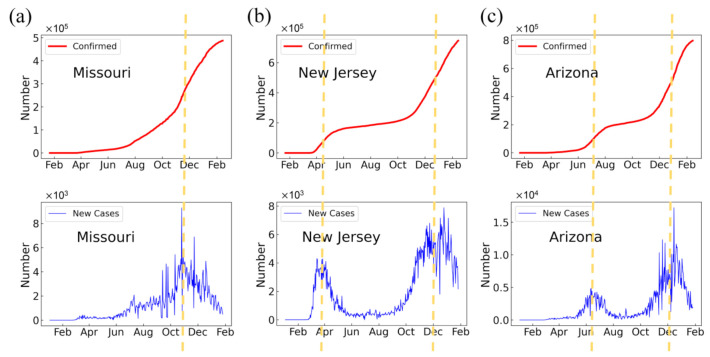
The daily confirmed cases (upper row) and corresponding new cases (lower row) of COVID-19 for three selected states: Missouri (**a**), New Jersey (**b**), and Arizona (**c**), in the US that represent three categories (one outbreak; two outbreaks, with first outbreak in March–April; and two outbreaks, with first outbreak in June–August, respectively) of transmission dynamics in the US, where the dashed yellow lines indicate the epidemic outbreak peaks.

Figures S1 and S2 show the graphs of the daily numbers of confirmed cases and new cases with respect to the time period of 22 January 2020 to 14 February 2021 for each of the 50 US states, where the daily new cases were obtained by the confirmed cases of the current day minus those of the previous day. It can be seen from the figures that all states experienced at least one COVID-19 outbreak during the period. During a COVID-19 outbreak, the number of daily confirmed cases increased rapidly at an increasing rate in the early stages, which corresponds to the increasing numbers of daily new cases. After the number of daily new cases reached a maximum, the increase of daily confirmed cases became slower until it reached a relatively stable state. Therefore, a COVID-19 outbreak showed an obvious peak on the curve of daily new cases and a significant plateau on the curve of daily confirmed cases following the outbreak (Figure 1).

**Figure 2:**
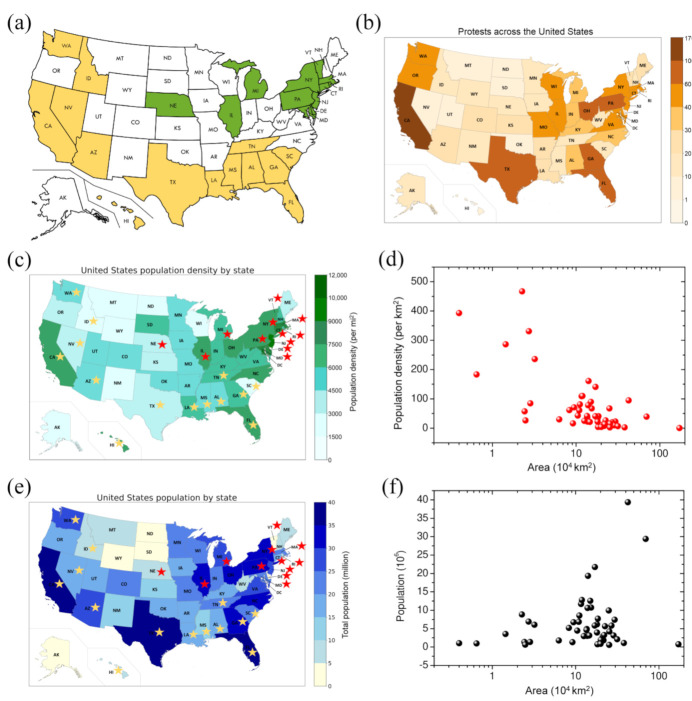
The color-coded maps of the US states. (**a**) The states with two epidemic outbreaks occurring in March–April and October–January are highlighted in green, and those with two epidemic outbreaks occurring in June–August and October–January are highlighted in yellow. (**b**) Protests across the US by the number of cities and towns with rallies or protests in a state, the data for which were taken from https://www.usatoday.com/in-depth/graphics/2020/06/03/map-protests-wake-george-floyds-death/5310149002. (**c**) Population densities and (**e**) populations of the states with two epidemic outbreaks occurring in March–April and October–January are indicated by a red star, and those with two epidemic outbreaks occurring in June–August and October–January are indicated by a yellow star. (**d**) The population densities vs. areas for the 50 states in the US. (**f**) The populations vs. areas for the 50 states in the US.

From Figures S1 and S2, one can also see that all the outbreaks occurred in one of the three different time windows (Figure 1). The first time window for COVID-19 outbreaks was March–April, 2020, example states for which include Connecticut, Delaware, Maryland, Massachusetts, and New Jersey (Figure 2a). The main reason for the outbreak during this time period may be due to the high infectivity of COVID-19 in the cold weather because the virus survives longer and has a higher infectious ability at lower temperatures [45]. In addition, part of the outbreak in this time window may have been caused by the early introduction of the coronavirus to these states with little to no measures enacted to control the spread because in the early stages, both the individuals and the government had little knowledge about the virus and were not aware of the high risk of the virus. The period between the first and second outbreaks where the spread of the coronavirus becomes relatively stable is the result of warmer weather in which the disease experiences a loss in the reproduction rate as well as the enacted measures to prevent the spread of the disease. The second time window for COVID-19 outbreaks is June–August 2020. Examples of states that underwent an outbreak during the period include Alabama, Arizona, California, Florida, and Texas (Figure 2a). Theoretically, an outbreak was not expected to occur during this summer period because the infectious ability of the virus decreases at a higher temperature. However, the outbreaks occurred due to human behavior more than the infectivity of the virus. This is indeed the case because several “Black Lives Matters (BLM)” protests occurred in many states during this period of time. These protests may have led to the fast spread of the COVID-19 virus due to large gatherings of people. As shown in Figure 2a,b, those states with large and extreme protests are likely to have an outbreak during this time, which confirms the important impact of protests on the COVID-19 outbreak. Additionally, summer break, which caused a higher mobility of people and lower awareness of the coronavirus, resulting in less incentive to maintain preventive measures, may have played a role. As shown in Figure 2a, many states with this outbreak have coastline access to the ocean or neighbor coastal states. As such, these states would involve more gatherings of people due to vacations, tours, beach activities, etc., which favor the spread of COVID-19. The third time window for COVID-19 outbreaks is October 2020–January 2021. Interestingly, all states show a COVID-19 outbreak during this time (Figures S1 and S2). The outbreak in this time widow could be attributed to both the infectious virus and human behavior. On one hand, the virus becomes more infectious at lower temperatures and has a higher transmission ability in cold weather. On the other hand, after a long period of fighting against the virus, individuals may tend to lower their sensitivity to the virus due to a lack of awareness and the relaxation of the public health interventions by governments due to economic reasons.

#### 3.1.2. Properties of COVID-19 Outbreaks

The transmission dynamics of COVID-19 are also state-dependent in terms of epidemic outbreaks in the US. Roughly, the 50 states can be grouped into two categories according to the number of COVID-19 outbreaks (Figures S1 and S2). One category is those with only one COVID-19 outbreak in October 2020–January 2021 (Figure 1a). Examples of states included in this category are Alaska, Missouri, and South Dakota (Figure S1). In these states, the number of confirmed cases shows a logistic growth similar to that of the classic SIR model, which corresponds to a single peak in the curve of daily new cases. The other category is those with two outbreaks (Figure 1b,c). Examples of states included in this category are New Jersey, Arizona, and Massachusetts. The number of daily confirmed cases grows logistically then flattens out into a linear growth for a period of time and then grows logistically once again, which corresponds to two major peaks in the curves of daily new cases.

The category of states with two outbreaks can also be further divided into two subcategories. The states in these two subcategories all had a second outbreak in October 2020–January 2021 but differ in the time of the first outbreak. One subcategory had the first outbreak in March–April 2020 (Figure 1b). Examples of states included in this subcategory include Connecticut, Massachusetts, New Jersey, and New York. The other subcategory had the first outbreak in June–August 2020 (Figure 1c). Examples of states included in this subcategory are Alabama, Arizona, California, Florida, and Texas. Interestingly, a few states like Hawaii, Louisiana, and Maryland seem to show three COVID-19 outbreaks, with outbreaks occurring in March–April, June–August, and October–January 2021. Nevertheless, they may be roughly grouped into the two-outbreak category due to one outbreak having much less impact than the other two. Given such two-outbreak properties of the US states, where one outbreak can also be effectively regarded as two outbreaks (i.e., one is null and the other is real), we will be able to model the transmission dynamics of COVID-19 in the 50 states of the US using our two-outbreak epidemic model shown in Equations (1) and (2).

#### 3.1.3. Demographic Impact on COVID-19 Outbreaks

Given that the 50 states of the US have very different populations and areas, it would be valuable to examine how such geographical and/or demographic features impact the spread of COVID-19. Table 1 highlights those two-outbreak states in ranked lists of the states according to their population densities, populations, and areas. It can be seen from the table that, overall, those states with higher populations or population densities tend to have two outbreaks. It can be understood because the higher population density causes the virus to be easier to spread due to the smaller distance between individuals and the larger population, leading to a higher possibility for the virus to spread due to the fact that there are more exposed people.

Further examination of two-outbreak states also reveals that those states with higher population densities tended to have an outbreak in March–April 2020, while states with higher populations tended to have an outbreak in June–August 2020. As shown in Table 1, the states with the top seven population densities all had two outbreaks, with the first outbreak occurring in March–April (Table 1 and Figure 2c), and the states with the top three populations all experienced two outbreaks, with the first outbreak occurring in June–August (Table 1 and Figure 2e). This kind of demography-dependent phenomenon is also understandable. As mentioned above, the outbreak in March–April is mostly due to the infectivity of the COVID-19 virus in cold weather. Therefore, it makes sense that the outbreaks in March–April tended to occur in those states with a high population density because the virus would be easier to spread due to the closer distance between individuals. In contrast, the outbreaks in June–August is mainly attributed to human behavior like BLM protests and high social mobility. For a state with a higher population, there would be more possible protests and higher social mobility due to the larger number of people, which explained the outbreaks in June–August, 2020.

Interestingly, the two-outbreak feature also seems to correlate with the total land area of the state. Namely, the outbreaks in March–April tended to occur in states with smaller areas, while the outbreaks in June–August tended to occur in states with larger areas (Table 1). However, it should be noted that such an area-dependent phenomenon on the two-outbreak feature is not truly due to the total area of the state, but rather due to an indirect effect of population density. As shown in Figure 2, small-area states tend to have a high population density (Figure 2d), and large-area states tend to have a large population (Figure 2f). In addition, some states with low population density such as Nebraska, Nevada, and Idaho, also had two outbreaks. For Nevada and Idaho, we can see that the first outbreak for these two states occurred in July. Figure 2 shows that these two states are the neighbors of large-population states like California and Washington, which had many BLM protests, explaining the increase in transmission. As for Nebraska, although it shows an increase of daily confirmed cases in March-April, the outbreak was relatively weak, which would be due to its overall low population density. These findings are expected to provide valuable guidance for COVID-19 vaccine prioritization in the US.

### 3.2. Modeling the COVID-19 Dynamics of the US States

#### 3.2.1. Fitting of the T-SIR Model

**Figure 3:**
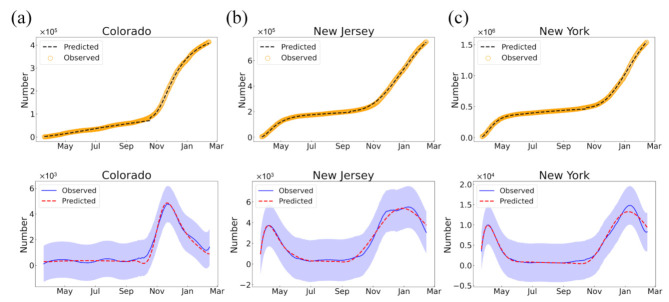
The fitting of our T-SIR model to the daily confirmed cases (upper row) and corresponding new cases (lower row) of COVID-19 for three selected states: Colorado (**a**), New Jersey (**b**), and New York (**c**) in the US that represent three categories of transmission dynamics in the US, where the dashed lines indicate the predicted data of T-SIR model. Data were smoothed using a Savitzky–Golay filter for daily new cases [46], where the light-blue shade indicates the standard deviations of reported data.

Based on our two-outbreak T-SIR epidemic model in Equations (1) and (2), we have modeled the transmission dynamics of COVID-19 in the 50 US states through the fitting of the T-SIR model to the daily confirmed cases from 22 January 2020 to 14 February 2021, where the compartments I and R of the model are regarded as the confirmed cases. It can be seen from the figures that overall, our T-SIR model fits the dynamics of the daily confirmed COVID-19 cases very well and is consistent with the data from most of the states (Figures S3 and S4). The model not only fits the COVID-19 dynamics of two-outbreak states like Alabama, Connecticut, Massachusetts, New Jersey, and New York, but also describes the epidemic trends of one-outbreak states like Alaska, Colorado, Kentucky, Ohio, and West Virginia (Figure 3a–c). Similar consistency between the predicted and real data can also be observed in the fitting of daily new cases, demonstrating the robustness of our T-SIR model (Figure 3). Nevertheless, for a few states, such as Hawaii, Iowa, and Louisiana, our T-SIR model seemed to not fit the epidemic dynamics of COVID-19 very well (Figures S3 and S4), which may be due to the multiple waves of the epidemic dynamics in these states and/or inherent limitations in the reported data.

#### 3.2.2. Implications of T-SIR Model Parameters

Through the model fitting, we can obtain the epidemic parameters of our T-SIR model, $\beta_{1}$, $\beta_{2}$, $\sigma_{1}$, $\sigma_{2}$_,_ and γ, for the 50 states of the US, where $\beta_{1}$ and $\sigma_{1}$ are for the first outbreak of COVID-19 occurring in March–April or June–August, and $\beta_{2}$ and $\sigma_{2}$ are for the second outbreak occurring in October–January. These epidemic parameters gave a quantitative description of the COVID-19 transmission dynamics of different states and will enable the investigation of the impact of state geographic and demographic data on the spread of the disease. Such investigations will provide a deep understanding of the epidemic dynamics of COVID-19 in different states and help the government create the corresponding measures to prevent the spread of COVID-19. As such, we have investigated the relationship between the epidemic parameters and the demographic data for the 50 US states. Specifically, the epidemic parameters for fitting the model using respective state geographic and demographic data were compiled. Pairwise comparisons were made by examining their Pearson correlations. A significant correlation between two different variables corresponds to an important linear relationship between the two factors.

**Figure 4:**
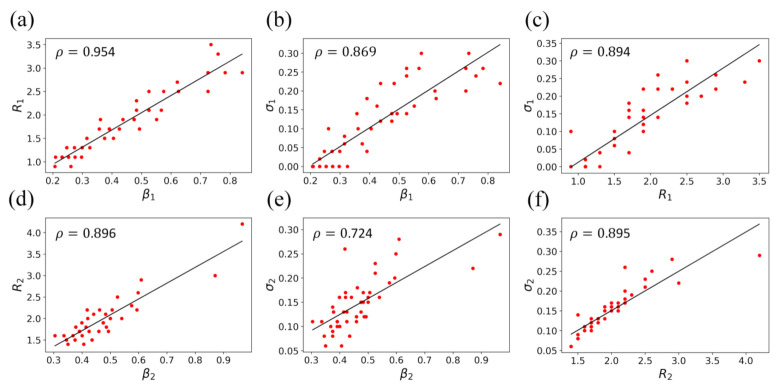
The pairwise relationships and their correlation coefficients of three epidemic parameters, R, β, and σ, of the T-SIR model for the first outbreak (**a**–**c**) and second outbreak (**d**–**f**), where the solid lines are the linear fittings of the data.

Figure 4a–c shows the relationships among the epidemic parameters, $\beta_{1}$, $\sigma_{1}$, and $R_{1}=\beta_{1}/\gamma$, for the first outbreak. High correlations can be seen among three pairs of parameters, which give a correlation coefficient of 0.954 for $R_{1}$ vs. $\beta_{1}$, 0.869 for $\sigma_{1}$ vs. $\beta_{1}$, and 0.894 for $\sigma_{1}$ vs. $R_{1}$. Given the relationship of $R_{1}=\beta_{1}/\gamma$, the high correlation between the reproduction number $R_{1}$ and transmission rate $\beta_{1}$ suggests that the removal rate γ is relatively constant for different states (Figure 4a). This means that people infected by the virus take about the same time to recover in different states, which is consistent with the general findings for infectious diseases. It is known that the invention parameter σ is an indicator of the government’s preventive measures and individuals’ self-protection to contain the coronavirus. A higher σ corresponds to more effective preventive measures. It can be seen from Figure 4b that there is a high correlation between $\sigma_{1}$ and $\beta_{1}$. This relationship can be understood because both individuals and the government adopt more and stricter preventive measures when the transmission rates are higher. These preventive measures may be both mandatory and voluntary, as higher caution is taken when large numbers of people are becoming infected. Figure 4c also shows a high correlation between $\sigma_{1}$ and $R_{1}$. This relationship is due to $\sigma_{1}$ being positively related to $\beta_{1}$. , and $\beta_{1}$ being positively correlated $R_{1}$ in the formulaic relationship $R_{1}=\beta_{1}/\gamma$, where the γ is relatively a constant. Similar relationships exist among the epidemic parameters, $\beta_{2}$, $\sigma_{2}$, and $R_{2}=\beta_{2}/\gamma$, for the second outbreak can also be observed, which give a correlation coefficient of 0.896 for $R_{2}$ vs. $\beta_{2}$, 0.724 for $\sigma_{2}$ vs. $\beta_{2}$, and 0.895 for $\sigma_{2}$ vs. $R_{2}$ (Figure 4d–f). The reasoning for these relationships is the same as that of the first outbreak.

**Figure 5:**
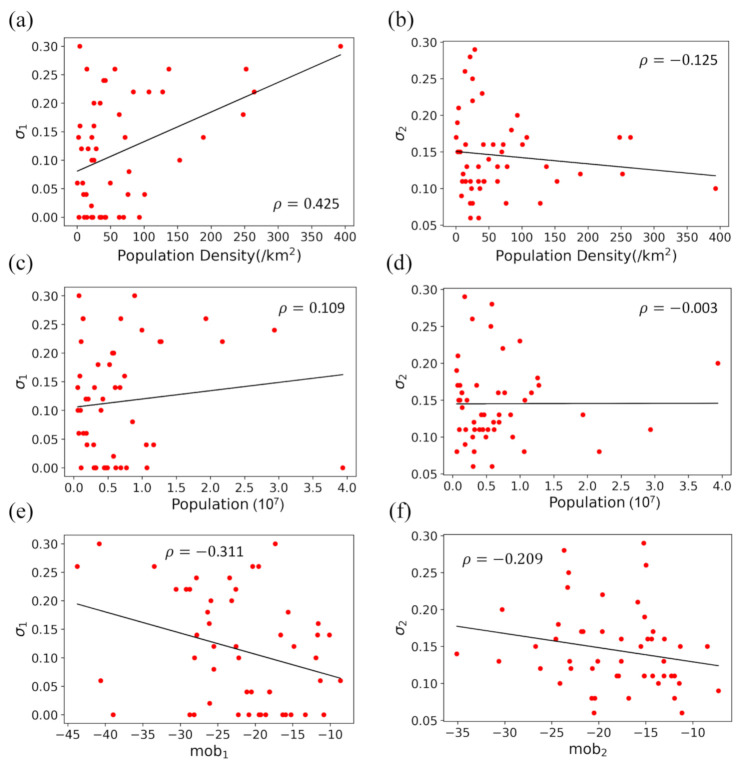
The intervention parameter σ vs. population density (**a**,**b**), population (**c**,**d**), and social mobility (**e**,**f**) for the 50 states in the US for the first and second outbreaks, respectively. The solid lines are the linear fittings of the data and the corresponding correlation coefficients are also shown.

As the intervention parameter, σ, is an indicator of the response of the government and individuals to the epidemic outbreak and thus the most important parameter of our T-SIR model, we will focus the investigation on the σ parameter. Figure 5a,b show the relationships between the σ parameters and the population densities of the 50 US states for the first and second outbreaks, respectively. It can be seen from the figure that $\sigma_{1}$ has a positive correlation of ρ=0.425 with the population density for the first outbreak but shows a weakly negative correlation of ρ=−0.125 with the population density for the second outbreak. The different relationships for the two outbreaks may be understood because σ is the overall effect of both government interventions and individuals’ self-protection measures. During the first outbreak, the COVID-19 virus was new to the government and individuals. In this case, both the government and individuals in states with higher population densities tended to take more preventive measures to contain the coronavirus. For the second outbreak, the negative correlation between $\sigma_{2}$ and population density would be due to relaxation of preventive measures for the virus, which resulted in an apparently lower σ for states with higher population densities because the virus spread more easily in higher-density populations if no strict interventions were enforced. Compared to the case made for population density, σ shows a weaker relationship with the population (Figure 5c,d). This is understandable because it is the population density rather than the population that determines the spread of a disease in a normal epidemic.

We further investigated the relationship between σ and social mobility, where the mobility during the first outbreak is roughly taken from the average over Mach–June and that for the second outbreak is taken from the average over October–January. As shown in Figure 5e, a significant negative correlation of ρ=−0.311 between $\sigma_{1}$ and mobility can be seen for the first outbreak. This is explained by a higher σ indicating that the government enforced more preventive measures. Measures to control the spread of the disease including social distancing and stay-at-home isolation orders decreased people’s mobility. However, a lower correlation of ρ=−0.209 is observed for the second outbreak (Figure 5f). This may be understood because the effect of σ comes from two parts: government interventions and individual self-protection measures, where government interventions have a much higher impact on social mobility than the self-imposed measures that individuals place on themselves. With the relaxation of government interventions towards the second outbreak, σ came more from the less impactful individual self-protection measures, which explains the smaller correlation between $\sigma_{2}$ and mobility.

**Figure 6:**
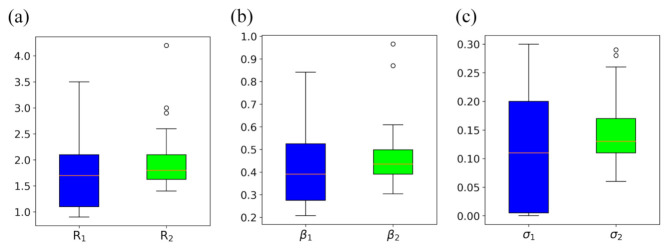
The comparison between the three parameters of the T-SIR model for the first outbreaks (blue) and second outbreaks (green) of the 50 US states. (**a**) Reproduction numbers R. (**b**) Transmission rates β. (**c**) Intervention parameters σ.

In addition, we also compared the magnitudes of the epidemic parameters, R, β, and σ, of the first and second outbreaks, which are shown in Figure 6. It can be seen from the figure that the initial transmission rates $\beta_{1}$ and corresponding reproduction numbers $R_{1}$ for the first outbreak have a wider range than those of the second outbreak. This is understandable because people knew little about COVID-19 at the beginning, and the government had not enforced intervention measures to prevent the transmission of the virus yet, which would lead to a high transmission rate (Figure 6a). At the same time, some states did not show an outbreak during this early stage due to little introduction of COVID-19, which yielded a low transmission rate. However, after the epidemic had gone on for a few months, both the government and individuals all had a better understanding of COVID-19 and adopted more efficient prevention measures to stop the spread of the virus in the late stages of the COVID-19 epidemic, resulting in relatively moderate transmission rates for the second outbreak (Figure 6b). Similarly, as the government and individuals adopt the corresponding preventive measures to contain the virus according to the transmission rates, the range of σ will be similar to that of β, resulting in an overall wider range of σ for the first outbreak than for the second outbreak (Figure 6c).

## 4. Discussion

Based on our time-dependent T-SIR model, we have modeled the transmission dynamics of COVID-19 from 22 January 2020 to 14 February 2021 in the 50 states of the US. The predicted and reported data are well consistent in both the daily confirmed cases and new cases, suggesting the robustness of our T-SIR model. Nevertheless, there may be limitations in the reported COVID-19 data because the availability of the reported data depends on various factors. First, the availability of confirmed data strongly depends on COVID-19 testing capacity. This issue was especially critical in the early stages of the epidemic when the number of infected individuals was far beyond the testing capacity for COVID-19 capacity. Second, the testing capacity is also state or city-dependent due to the differences in the hospital resources in different states or cities. Moreover, many people might not go to the hospital due to having mild or no symptoms even if they were infected, and thus these infected individuals would not be counted. In addition, compared to the confirmation of infected individuals, it is much more difficult to track recovered individuals. Therefore, it is difficult to accurately separate infected and recovered individuals in the confirmed cases. These issues in the reported data significantly limit the accurate modeling of COVID-19 epidemic dynamics, especially for the dynamics of daily new cases (Figure S4), though the basic trends can be modeled.

Despite the various limitations in the data of reported COVID-19 cases, our T-SIR model still modeled the time-dependent transmission dynamics of the virus for the 50 states of the US well, demonstrating its robustness to model the impact of non-pharmaceutical interventions on the spread of COVID-19. In other words, our T-SIR model truly reflects the unique epidemic dynamics of COVID-19 in the US. In our model, the transmission rate, $\beta \left(t\right)$, reduces quickly in the early stages and then changes more slowly as time passes. This is consistent with the social scenario of individuals and public health responses to an outbreak of COVID-19 in the US. During the early stages of an outbreak, the number of new cases and deaths increased rapidly, which resulted in strong pressure on society for prevention. Therefore, during this period, both the individuals and government had a tendency to adopt strict measures and/or interventions to reduce the transmission of the disease, resulting in a fast-decreasing transmission rate. When the COVID-19 epidemic entered the later stages of an outbreak after some time, the transmission rate became lower and the epidemic seemed to be under control. Correspondingly, the epidemic entered a relatively stable stage. During this stage, both the individuals and government had a tendency to relax the measures and/or interventions because of less pressure from the epidemic and economic considerations, which would result in a second epidemic outbreak, as shown in the dynamics of COVID-19 (Figures S1 and S2).

Although there is good consistency between the reported data of COVID-19 and the predicted values by our T-SIR model, it should be noted that the reported data gather only the infected people controlled by the health system, as mentioned before. These controls depend on the number of tests performed on people. For instance, people with symptoms who are not controlled for the system and the asymptomatic are not considered, although they are also infected. Therefore, the reported data are only a fraction of real infected cases. These inherent factors may need to be considered when interpreting the present T-SIR and other epidemic models.

In addition, it is known that vaccination is the ultimate measure to fight against COVID-19 due to the fact that there are no effective drugs for the treatment of the disease [47]. Starting from around December 2020, an increasing number of people around the world began to have the opportunity to receive COVID-19 vaccines. In the US, the percentage of the population that is fully vaccinated against COVID-19 increased quickly from 0.48% on 15 January 2021, to 23.47% on 15 April 2020, to 46.31% on 30 June 2021 [48]. The share of people who had received at least one dose of the COVID-19 vaccine increased from 3.17% on 15 January 2021, to 37.62% on 15 April 2020, to 54.02% on 30 June 2021 [48]. The increasing number of vaccinated people play an important role in stopping the transmission of COVID-19, which may in part explain the decreasing new cases of COVID-19 after January 2021 in all US states (Figures S1 and S2).

## 5. Conclusions

We have investigated the transmission dynamics of COVID-19 from 22 January 2020 to 14 February 2021 in the 50 states of the US and also proposed a time-dependent Susceptible-Infectious-Removed model, named T-SIR, to model the epidemic dynamics of the virus. It was revealed that the 50 states can be roughly divided into two groups according to the number of occurred epidemic outbreaks: the states with one outbreak and the states with two outbreaks. Those states with higher populations and/or population densities tend to have a higher chance of two COVID-19 outbreaks than the other states. Further examinations revealed that the states with high population density tend to have their first outbreak occur in March–April, which is mostly due to the high infectivity of COVID1-9 in the cold weather, while the states with a high population tended to have their first outbreak occur in June–August, which may be mostly due to protests and gatherings. All states had a COVID-19 outbreak in October–January, which would be attributed to the relaxation of both governmental intervention policies and individual self-prevention measures as well as the colder weather. We further fitted our T-SIR epidemic model to the transmission dynamics of COVID-19 in the 50 states of the US. It was shown that our T-SIR model can well describe the epidemic dynamics of all 50 states. The relationships between the corresponding epidemic parameters and demographic data were extensively investigated, providing insights into the spread of COVID-19 in different states. The present study will be valuable for not only understanding the epidemic dynamics of COVID-19 but also helping the government make effective virus-control policies such as mask wearing and vaccine prioritization in the US.

## Figures and Tables

**Table 1:** The ranked lists of the 50 states in the US according to their population densities, populations, and areas, where the states with two epidemic outbreaks occurring in March–April and October–January are highlighted in green, those with two epidemic outbreaks occurring in June–August and October–January are highlighted in yellow, and those with only one epidemic outbreak occurring in October–January are not highlighted.

Population Density			Population			Area		
Rank	State	Density (/km^2^)	Rank	State	Population	Rank	State	Area (km^2^)
1	New Jersey	393.18	1	California	39,368,078	1	Alaska	1,723,337
2	Rhode Island	264.22	2	Texas	29,360,759	2	Texas	695,662
3	Massachusetts	252.18	3	Florida	21,733,312	3	California	423,967
4	Connecticut	247.75	4	New York	19,336,776	4	Montana	380,831
5	Maryland	188.47	5	Pennsylvania	12,783,254	5	New Mexico	314,917
6	Delaware	153.09	6	Illinois	12,587,530	6	Arizona	295,234
7	New York	136.85	7	Ohio	11,693,217	7	Nevada	286,380
8	Florida	127.61	8	Georgia	10,710,017	8	Colorado	269,601
9	Pennsylvania	107.17	9	North Carolina	10,600,823	9	Oregon	254,799
10	Ohio	100.72	10	Michigan	9,966,555	10	Wyoming	253,335
11	California	92.86	11	New Jersey	8,882,371	11	Michigan	250,487
12	Illinois	83.92	12	Virginia	8,590,563	12	Minnesota	225,163
13	Virginia	77.54	13	Washington	7,693,612	13	Utah	219,882
14	North Carolina	76.05	14	Arizona	7,421,401	14	Idaho	216,443
15	Indiana	71.61	15	Massachusetts	6,893,574	15	Kansas	213,100
16	Georgia	69.59	16	Tennessee	6,886,834	16	Nebraska	200,330
17	Tennessee	63.09	17	Indiana	6,754,953	17	South Dakota	199,729
18	South Carolina	62.92	18	Missouri	6,151,548	18	Washington	184,661
19	New Hampshire	56.43	19	Maryland	6,055,802	19	North Dakota	183,108
20	Hawaii	49.69	20	Wisconsin	5,832,655	20	Oklahoma	181,037
21	Kentucky	42.78	21	Colorado	5,807,719	21	Missouri	180,540
22	Texas	42.21	22	Minnesota	5,657,342	22	Florida	170,312
23	Washington	41.66	23	South Carolina	5,218,040	23	Wisconsin	169,635
24	Michigan	39.79	24	Alabama	4,921,532	24	Georgia	153,910
25	Alabama	36.25	25	Louisiana	4,645,318	25	Illinois	149,995
26	Wisconsin	34.38	26	Kentucky	4,477,251	26	Iowa	145,746
27	Louisiana	34.24	27	Oregon	4,241,507	27	New York	141,297
28	Missouri	34.07	28	Oklahoma	3,980,783	28	North Carolina	139,391
29	West Virginia	28.44	29	Connecticut	3,557,006	29	Arkansas	137,732
30	Arizona	25.14	30	Utah	3,249,879	30	Alabama	135,767
31	Minnesota	25.13	31	Iowa	3,163,561	31	Louisiana	135,659
32	Vermont	25.03	32	Nevada	3,138,259	32	Mississippi	125,438
33	Mississippi	23.65	33	Arkansas	3,030,522	33	Pennsylvania	119,280
34	Arkansas	22.00	34	Mississippi	2,966,786	34	Ohio	116,098
35	Oklahoma	21.99	35	Kansas	2,913,805	35	Virginia	110,787
36	Iowa	21.71	36	New Mexico	2,106,319	36	Tennessee	109,153
37	Colorado	21.54	37	Nebraska	1,937,552	37	Kentucky	104,656
38	Oregon	16.65	38	Idaho	1,826,913	38	Indiana	94,326
39	Utah	14.78	39	West Virginia	1,784,787	39	Maine	91,633
40	Maine	14.73	40	Hawaii	1,407,006	40	South Carolina	82,933
41	Kansas	13.67	41	New Hampshire	1,366,275	41	West Virginia	62,756
42	Nevada	10.96	42	Maine	1,350,141	42	Maryland	32,131
43	Nebraska	9.67	43	Montana	1,080,577	43	Hawaii	28,313
44	Idaho	8.44	44	Rhode Island	1,057,125	44	Massachusetts	27,336
45	New Mexico	6.69	45	Delaware	986,809	45	Vermont	24,906
46	South Dakota	4.47	46	South Dakota	892,717	46	New Hampshire	24,214
47	North Dakota	4.18	47	North Dakota	765,309	47	New Jersey	22,591
48	Montana	2.84	48	Alaska	731,158	48	Connecticut	14,357
49	Wyoming	2.30	49	Vermont	623,347	49	Delaware	6,446
50	Alaska	0.42	50	Wyoming	582,328	50	Rhode Island	4,001

## Data Availability

The data used for this study are available at https://github.com/HuangDerek/T-SIR (accessed on 3 July 2021) or from the corresponding authors upon reasonable request.

## Supplementary Materials

The following are available online at https://www.mdpi.com/article/10.3390/ijerph18147594/s1, Figure S1: The daily confirmed cases of COVID-19 from 22 January 2020 to 14 February 2021 for the 50 states in the US, Figure S2: The daily new cases of COVID-19 from 22 January 2020 to 14 February 2021 for the 50 states in the US, where the data were calculated from the confirmed data, Figure S3: The fitting of our T-SIR model to the daily confirmed cases of COVID-19 for the 50 states in the US, where the dashed lines indicate the predicted data of T-SIR model, Figure S4: The fitting of our T-SIR model to the daily new cases of COVID-19 for the 50 states in the US, where the dashed lines indicate the predicted data of T-SIR model. Data were smoothed using a Savitzky–Golay filter for the daily new cases.
