# Supplementary material for: Modeling of the Long-Term Epidemic Dynamics of COVID-19 in the United States
Source: Int J Environ Res Public Health. 2021 Jul 16;18(14):7594. doi: 10.3390/ijerph18147594 (PMC8305610; doi:10.3390/ijerph18147594)
Supplement: Supplementary file 1 [file ijerph-18-07594-s001.zip › ijerph-1276371-supplementary.pdf]

Supplementary Materials

Modeling of the Long-Term Epidemic Dynamics of COVID-19 in the United States

Derek Huang [1], Huanyu Tao [2], Qilong Wu [2], Sheng-You Huang [2,*] and Yi Xiao [2,*]

[1] Wuhan Britain-China School, No.10 Gutian Rd., Qiaokou District, Wuhan 430022, China; huangderek04@gmail.com
[2] Institute of Biophysics, School of Physics, Huazhong University of Science and Technology, Wuhan 430074, China; d201980099@hust.edu.cn (H.T.); m201970198@hust.edu.cn (Q.W.)
* Correspondence: huangsy@hust.edu.cn (S.-Y.H.); yxiao@hust.edu.cn (Y.X.)

Figure S1

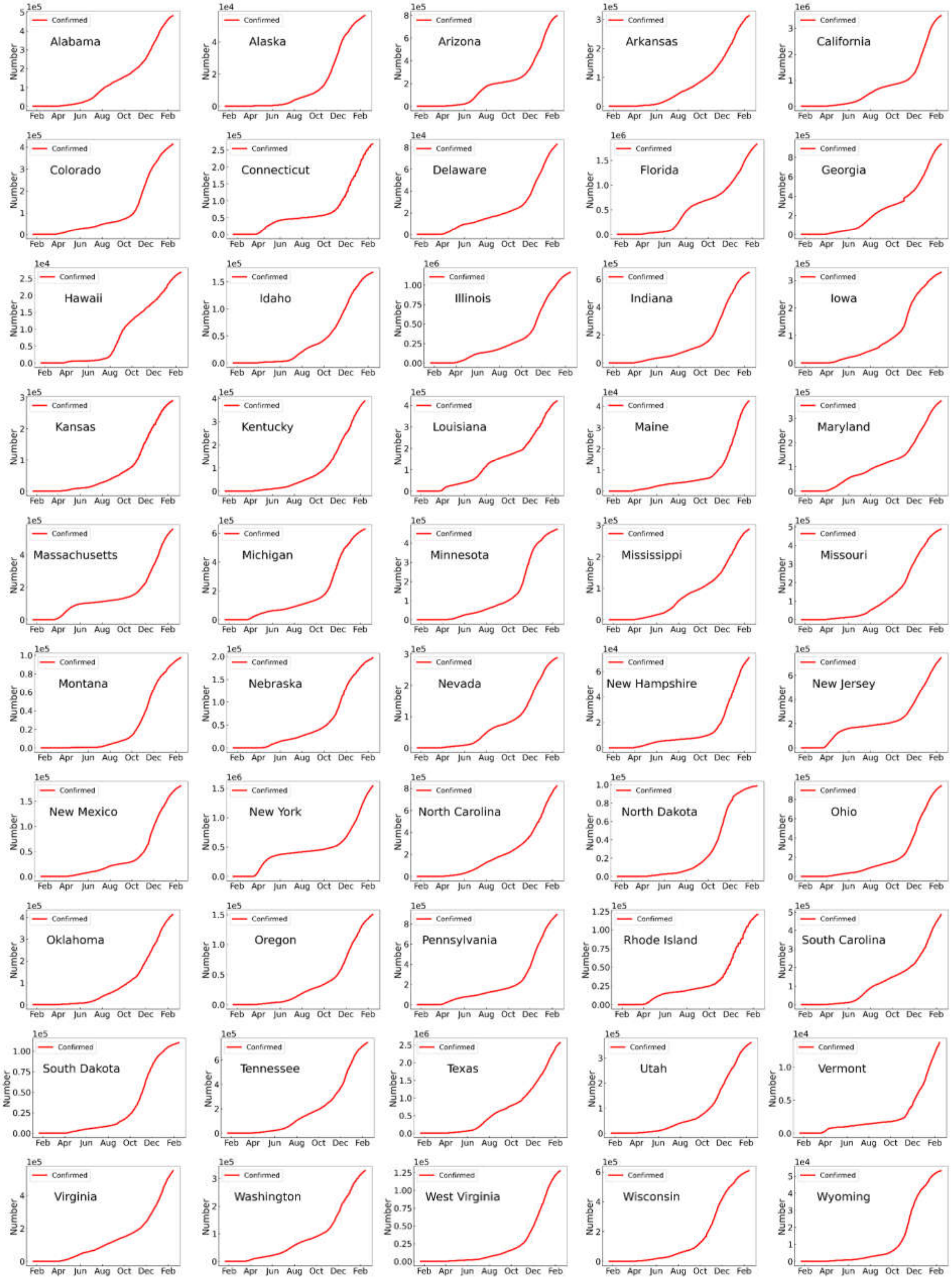

Figure S1: The daily confirmed cases of COVID-19 from Jan. 22nd, 2020 to Feb 14th, 2021 for the 50 states in the US.

Figure S2

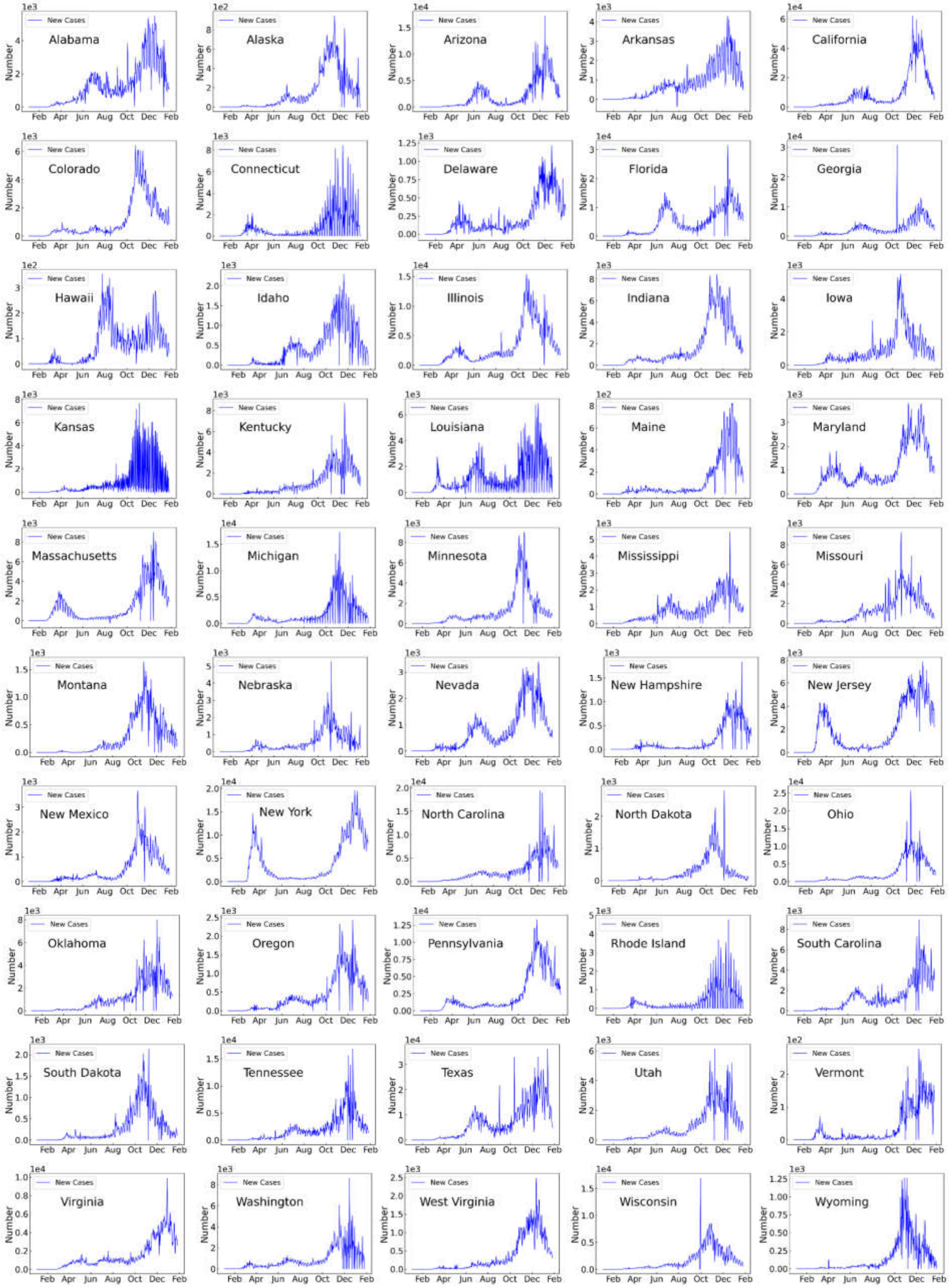

Figure S2: The daily new cases of COVID-19 from Jan. 22nd, 2020 to Feb 14th, 2021 for the 50 states in the US, where the data were calculated from the confirmed data.

Figure S3

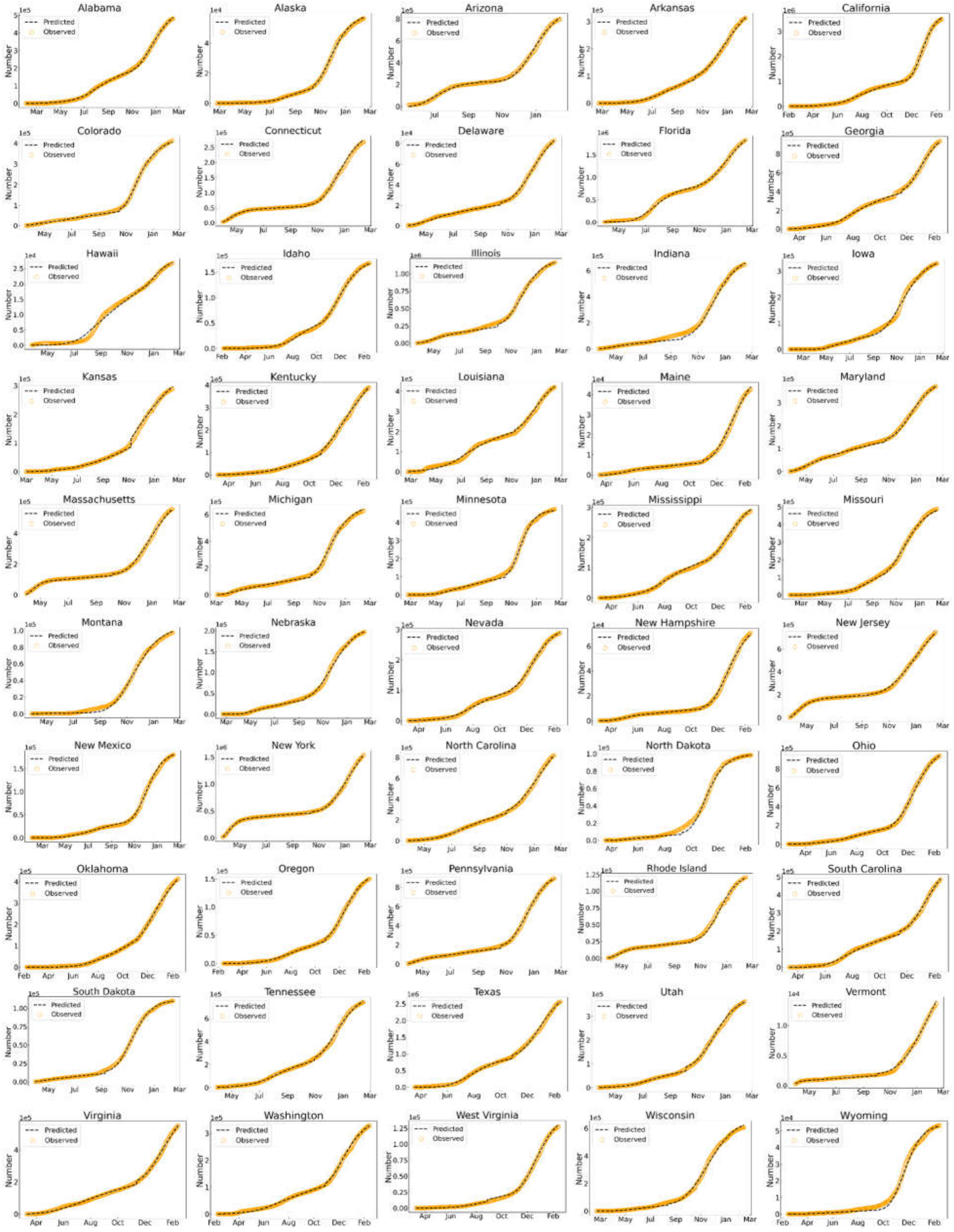

Figure S3: The fitting of our T-SIR model to the daily confirmed cases of COVID-19 for the 50 states in the US, where the dashed lines indicate the predicted data of T-SIR model.

Figure S4

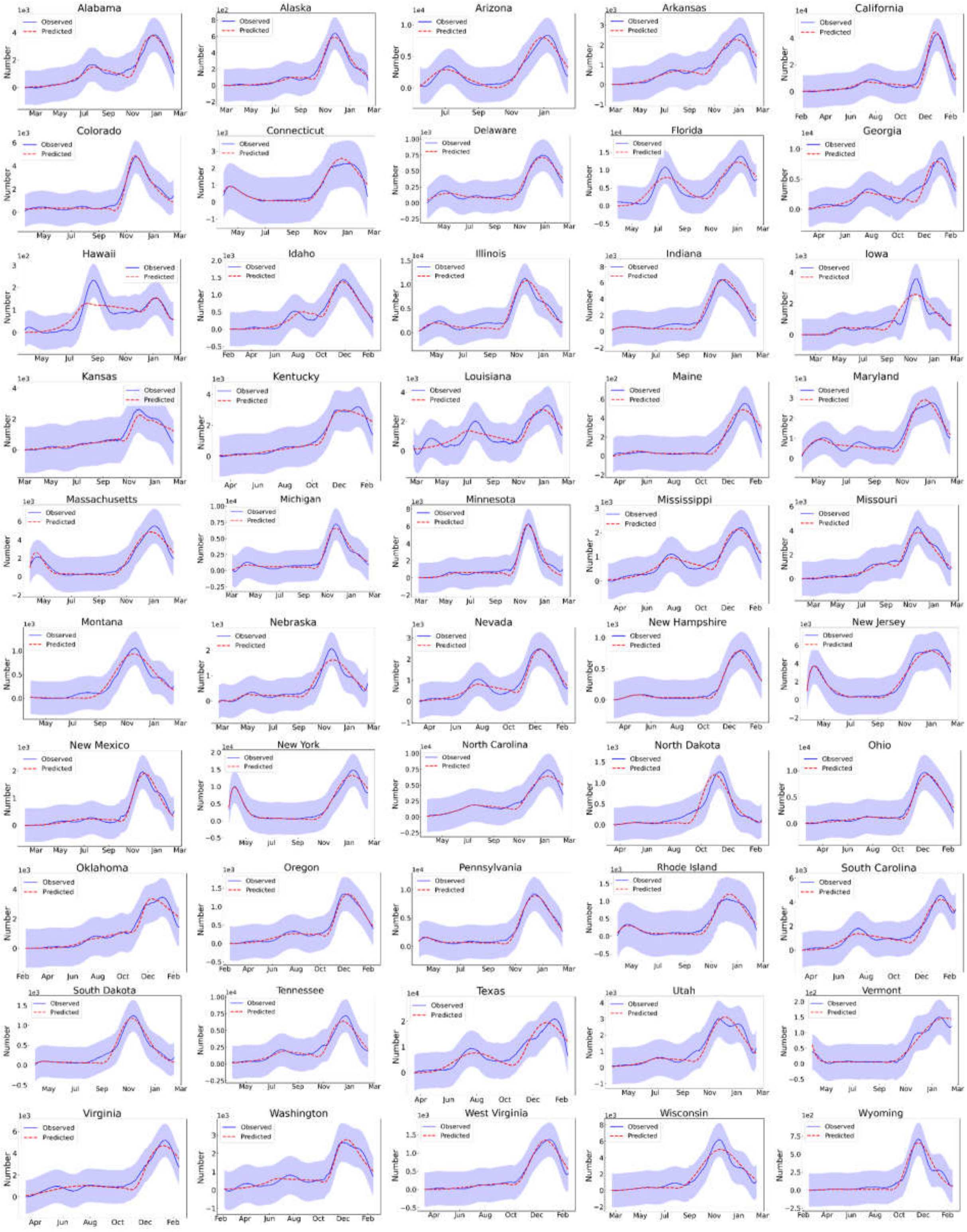

Figure S4: The fitting of our T-SIR model to the daily new cases of COVID-19 for the 50 states in the US, where the dashed lines indicate the predicted data of T-SIR model. Data were smoothed using a Savitzky–Golay filter for the daily new cases, where the light-blue shade indicates the standard deviations of reported data.
